# Prevalence of Dental Caries in 5- and 6-Year-Old Myanmar Children

**DOI:** 10.1155/2019/5948379

**Published:** 2019-04-28

**Authors:** Yoshiaki Nomura, Khin Maung, Eint Min Kay Khine, Khin Myo Sint, May Phyo Lin, Min Khaing Win Myint, Thu Aung, Kaoru Sogabe, Ryoko Otsuka, Ayako Okada, Erika Kakuta, Wit Yee Wint, Masahide Uraguchi, Ryo Hasegawa, Nobuhiro Hanada

**Affiliations:** ^1^Department of Translational Research, Tsurumi University School of Dental Medicine, 2-1-3 Tsurumi, Tsurumi-ku, Yokohama 230-8501, Japan; ^2^Oral Health Division, Department of Medical Services, Ministry of Health and Sports, Myanmar; ^3^Department of Operative Dentistry, Tsurumi University School of Dental Medicine, 2-1-3 Tsurumi, Tsurumi-ku, Yokohama 230-8501, Japan; ^4^Department of Oral Bacteriology, Tsurumi University School of Dental Medicine, 2-1-3 Tsurumi, Tsurumi-ku, Yokohama 230-8501, Japan; ^5^Department of Pediatric Dentistry, Tokyo Medical and Dental University, 1-5-45 Yushima, Bunkyo-ku, Tokyo 113-8510, Japan; ^6^INGO Association of Dental Volunteers of Japan, 1-2693 Nishiminatomachi-dori, Cyuou-ku, Niigata 951-8026, Japan

## Abstract

There are no national data available of the oral health in Myanmar. In this study, we examined dental caries status of 187 school children located in the suburban area of Naypyidaw, capital of Myanmar, at the age of five and six and analyzed by the individual level and tooth level. Maxillary D and B were sensitive for dental caries almost at the same level. They were less sensitive than maxillary A. Mandibular A and B were tolerant for dental caries. Prevalence of dental caries in Myanmar children was still high. By applying item response theory and multilevel modeling, tooth level analysis can be implemented to confirm the tendency for sensitivity or tolerance for dental caries by the tooth level.

## 1. Introduction

Decline of the prevalence of dental caries is a global trend [1]. However, in developing countries, prevalence of dental caries is still at a high level [2–5]. In Myanmar, more than 7% of economic growth was achieved in 2018 due to ease of economic constraints. The IMF predicted that this trend will continue in the future. However, there are large disparities in living standards. Even in the suburbs of the capital city of Myanmar, the living infrastructure centering on agriculture, water supply, and sewerage is still not promoted. Some people do not have tooth-brushing habit, and fluoride contained tooth paste is not widely used. Even though evidence-based dental caries programs are available in developed countries [6–10], in most of the areas in Myanmar, these programs are not yet applicable. However, under these conditions, accumulating data on oral hygiene is important to establish national policies for improving oral hygiene. However, under these conditions, accumulation of the data of oral health is important to establish national policy for improvement of oral health.

There are several national data available mainly in advanced countries; until now, there are no data available of the oral health in Myanmar. In this study, we examined dental caries status of school children located in the suburban area of Naypyidaw, capital of Myanmar, at the age of five and six and analyzed by the individual level and tooth level. In addition, which kind of teeth is tended to be affected by dental caries was also examined by applying multilevel analysis.

## 2. Methods

### 2.1. Study Design

Cross-sectional survey was carried out, to investigate the prevalence of dental caries in Myanmar children.

### 2.2. Setting

Two schools located in the suburban area of Naypyidaw district, capital of Myanmar, were randomly selected from the list of schools.

### 2.3. Participants

All the children of the two schools of grades 1 and 2 were the participants in the study. They were aged at 5 and 6.

### 2.4. Oral Examination

Oral examination was carried out by two calibrated dentists working at National Dental Hospital according to the WHO standard. They were trained under the WHO standard capitulated dentist at Niigata University which is one of the WHO collaboration centers. Dental caries status was recorded according to the WHO standard. Dental caries status of primary teeth was recorded as the following criteria: sound, caries, filled, missing due to caries, or exfoliation. Permanent teeth were recorded as follows: sound, dental caries, filled, or not erupted. No filling teeth were observed.

### 2.5. Statistical Methods

Statistical analysis was carried out by the subject's level and tooth level. Distributions of dental caries status were summarized by the subject level and tooth level. For the tooth level analysis, item response theory (IRT) was applied to calculate the item discriminations and item difficulties for each tooth kind. These parameters indicated susceptibility of developing dental caries and incidence of dental caries. IRT analysis was carried out by R software with irtoys package by using the following formula:

(1) $$\begin{array}{c} P\left(U_{i,j}=1\left|\right)\theta_{i},a_{j},b_{j}\right)=\frac{\exp\left(1.76a_{j}\left(\theta_{i}-b_{j}\right)\right)}{1+\exp\left(1.76a_{j}\left(\theta_{i}-b_{j}\right)\right)}. \end{array}$$

As each tooth nested in subjects, subjects and tooth constructed hierarchy structure. Multilevel analysis was carried out to calculate the sensitivity for dental caries by tooth kind. Gender and age were used for subject-level parameter, and tooth kind was used for tooth-level parameter. The statistical model was constructed by following model specification by IBM SPSS Statistics Ver 24.0 (IBM, Tokyo, Japan).

  Data Structure: subject, tooth
  Probability distribution: binominal
  Link function: probit

Fixed effects (resp., random effects or error terms) denoted by Greek letters (resp., alphabet) are given in the following model.

### 2.6. Model

Subjects, tooth, and tooth surface are indexed by *ijk*:

(2) $$\begin{array}{c} -L1:P_{o}{\left(de\;\;f\right)}_{ijk}=P_{o}\left(\mu_{jk}\right)=\pi_{0ijk}+\pi_{1jk}{\left(\text{subject}\right)}_{ijk}+\varepsilon_{ijk},\\ -L2:\;\;\pi_{0jk}=\;\;\beta_{00k}^{\left(m\right)}+\overset{3}{\underset{m=2}{\sum }}\beta_{001}^{\left(m\right)}{\left(\text{dental caries indexed by }m\right)}_{jk}+r_{0jk},\\ -L3:\;\;\pi_{1jk}=\beta_{10k},\\ \text{where dental caries}∼Bin\left(\alpha_{ijk},\tau_{ijk}\right)\text{ with }\mu_{ijk}=\frac{\alpha_{ijk}}{\tau_{ijk}\;\;},\\ \;\;e_{ijk}∼N\left(0,\delta_{e}^{2}\right),\;\;r_{0jk}∼N\left(0,\delta_{r}^{2}\right),\text{ and }u_{01k}∼N\left(0,\delta_{u^{\left(m\right)}}^{2}\right). \end{array}$$

### 2.7. Ethical Approval

Informed consent was obtained by each children before oral examination. This study was approved by the Ethical Committee of Tsurumi University School of Dental Medicine (Approval Number: 1624).

## 3. Results

**Figure 1:**
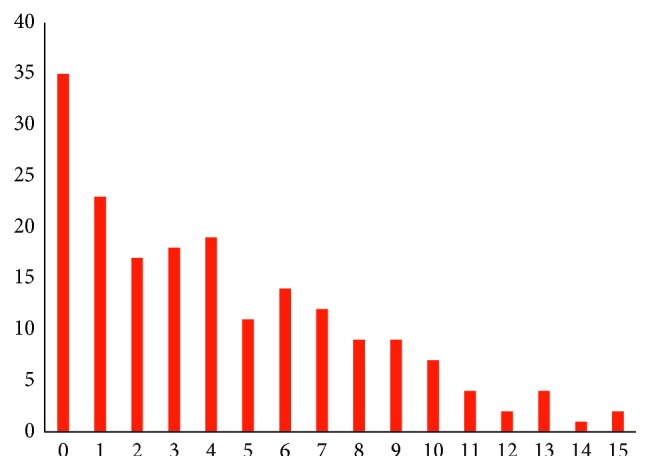
Distribution of dmf of the 187 Myanmar children aged 5 and 6. Even though the mode was 0, 152 (81.3%) subjects had at least one def tooth. The mean ± SD and median and 25th to 75th percentile were 4.26 ± 3.76 and 4 (1–7), respectively.

Distribution of dental caries in deciduous teeth (def) is shown in Figure 1. Among 187 subjects investigated in this study, 152 (81.3%) subjects had at least one def tooth and 35 (18.7%) were caries-free. Mean and standard deviation was 4.26 ± 3.76, and median and 25th to 75th percentile was 4 (1–7). Even though mode of def was 0, however, distribution was not skewed like Poisson distribution. When compared by gender and age, differences were not statistically significant by the Mann–Whitney *U* test (*P* = 0.670 and 0.949, respectively).

**Figure 2:**
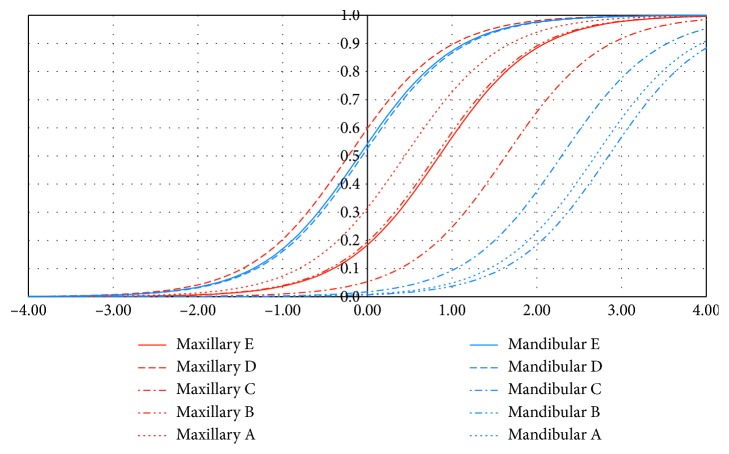
Item response curve of the sensitivity for dental caries of deciduous teeth. Horizontal axis indicates the total score of dental caries which is standardized as the def; 99% of def were transformed into −4 to 4. Vertical axis indicates the percent of subjects with dental caries for each tooth. When the ability (total score of dental caries) is fixed, prevalence of dental caries can be estimated. The curve located left side indicated that the teeth are highly susceptible to dental caries. The curve located right side indicated that the teeth are highly tolerant to dental caries.

Tooth-level distributions of def are shown in Tables 1 and 2. Prevalence of dental caries in deciduous teeth was varied. As many of the permanent teeth were not erupted, almost no dental caries were observed. Prevalence of dental caries was higher in molar tooth, maxillary E, and mandibular D and E. Prevalence of maxillary anterior teeth (A and B) were around 50%. However, even at such high prevalence of dental caries, prevalence of dental caries in mandibular anterior teeth was at low level. These tendencies were confirmed by item response theory (IRT). The results are shown in Table 3. The results of the item response curve are graphically illustrated in Figure 2. In this case, item discrimination (*a*_j_) indicated the slope of the item response curve, and item difficulty (*b*_j_) indicated the rising phase of the curve. Horizontal axis indicates the total score of dental caries which is the standardized def; 99% of def were transformed into −4 to 4. Vertical axis indicated the percent of subjects with dental caries for each tooth. When the ability (total score of dental caries) is fixed, prevalence of dental caries can be estimated. The curve located left side indicated that the teeth are highly susceptible to dental caries. The curve located right side indicated that the teeth are highly tolerant to dental caries. Maxillary D was the most sensitive for dental caries, and subsequently, mandibular D and E were sensitive for dental caries. Maxillary D and B were sensitive for dental caries almost at the same level. They were less sensitive than maxillary A. Mandibular A and B were tolerant for dental caries. Additionally, this tendency was confirmed by the multilevel modeling including age and gender as confounding. Mandibular anterior teeth were significantly tolerant when maxillary molar teeth were used as reference (Table 4).

## 4. Discussion

The caries status of permanent teeth in 12-year-old children has been documented in the database of the World Health Organization (WHO) [11]. However, little information is available about dental caries in primary dentitions. Population of Myanmar is more than 56 million people in 2018; however, a few reports are available for the dental caries status in primary dentitions [12, 13].

In this study, highly decayed teeth were observed in children (below 5 years) in Myanmar. A high proportion of decayed teeth with a low percentage of filled teeth suggest delays or barriers to receiving oral health promotion and oral healthcare services among the people in Myanmar, and this might be due to the insufficient dental professionals and lack of people's awareness on proper oral health. There are no dental hygienist systems in Myanmar. Additionally, financial hardship and geographical barriers may prevent people to reach service places in some remote areas.

The government has started the “School Health Program,” and the School Health Division of the Department of Health takes the main responsibility for planning and implementation of school health programs. Recently, the Oral Health Unit of the Department of Health of Myanmar introduced various oral health promotion activities and programs for different targeted groups to reduce oral disease and to promote community oral health status. For school children, “Early Childhood Caries Prevention Program” and “Institutional-based School Oral Healthcare Activity” have been implemented. These programs include correcting tooth-brushing activities in below 5-year-old children and giving oral health education to caregivers. However, due to the limited resources of dental professions, these programs carried out very limited area. In addition, “Feasible Effective and Affordable Fluoride Program” for good oral health for the whole population have been implemented. However, the tap water supply coverage ratio is still low. Many people still use well water except for the urban area. In some rural areas, people are self-sufficient and have no cash receipts. These people cannot afford to buy tooth paste. There are still many obstacles to promote oral health promotion in Myanmar.

In this study, among 187 subjects investigated in this study, 152 (81.3%) subjects had at least one def tooth, and 35 (18.7%) were caries-free. Mean and standard deviation was 4.26 ± 3.76, and median and 25th to 75th percentile was 4 (1–7). These figures were high levels among Southeast Asian countries [14].

The results in this study indicate that childhood dental caries is still a serious dental public health problem in Myanmar that needs attention by the government and policy makers. In Myanmar, tooth-brushing habits and fluoride-containing tooth paste were not widely spread. School programs including oral health education, tooth brushing instruction, and fluoride mouthrinse programs were not carried out. There are several evidence-based preventive programs for dental caries. However, these programs had not been implemented.

Conventional regression analysis used dependent variable for objective variable and independent variables for explanatory variable. Dependent variable and independent variable are different kinds of variables. dmf or DMF is summary of the number of decayed teeth, missing teeth, and filled teeth. Regression analysis for dmf or DMF as the objective variable by the number of decayed, missing, and filled teeth as the explanatory variable should not be organized. Item response theory (IRT) was first proposed in the field of psychometrics for the purpose of ability assessment. It is widely used in education to calibrate and evaluate items in tests, questionnaires, and other instruments and to score subjects on their abilities, attitudes, or other latent traits. Today, all major educational tests, such as the Scholastic Aptitude Test (SAT) and Graduate Record Examination (GRE), are developed by using item response theory.

In recent years, IRT-based models have also become increasingly popular in health outcomes, quality-of-life research, and clinical research [15–17]. However, the reports for oral health by IRT were limited [18, 19]. One of the properties of IRT is to be able to construct the formula of total score as the objective variable by items for total score as the explanatory variable. Therefore, IRT can be applicable for dmf or DMF as objective variable and dental caries status as explanatory variable. In addition, by using IRT, important item characteristics can be calculated. Item discrimination (*a_j_*) indicates how well an item can discriminate between subjects with different ability levels. Item discrimination is reflected in the steepness of the slope of the item characteristic curve. Item difficulty (*b_i_*) is reflected in the position of the item characteristic curve along the x-axis. It is generally considered that anterior incisor was tolerant for dental caries and molar teeth are sensitive for dental caries. In this study, we can confirm this tendency by applying IRT. Additionally, each tooth is nested in each individual; therefore, each tooth is not statistically independent. By applying multilevel modeling, we can overcome these problems. In addition to IRT, by the application of multilevel modeling, the tendency of sensitivity or tolerance at tooth level can be confirmed. In conclusion, prevalence of dental caries in Myanmar children was still at a high level. By applying item response theory and multilevel modeling, tooth-level analysis can be implemented to confirm the tendency for sensitivity or tolerance for dental caries by the tooth level.

## Figures and Tables

**Table 1:** Tooth-level distribution of dental caries in primary teeth.

	**55**	**54**	**53**	**52**	**51**	**61**	**62**	**63**	**64**	**65**	**85**	**84**	**83**	**82**	**81**	**71**	**72**	**73**	**74**	**75**
Sound	147	104	171	134	109	113	138	172	110	153	109	114	180	165	137	143	168	180	110	105
Caries	40	76	15	40	52	51	41	15	75	34	75	71	7	3	1	1	1	7	77	78
Filled	0	0	0	0	0	0	0	0	0	0	0	0	0	0	0	0	0	0	0	1
Missing due to caries	0	6	1	5	6	3	1	0	1	0	3	2	0	0	3	1	1	0	0	0
Exfoliation	0	1	0	8	20	20	7	0	1	0	0	0	0	19	46	42	17	0	0	0

Bold numbers indicate the tooth kind by the WHO standard code. Almost no treated teeth were observed. Most of the permanent teeth were not erupted.

**Table 2:** Tooth-level distribution of dental caries in permanent teeth.

	**17**	**16**	**15**	**14**	**13**	**12**	**11**	**21**	**22**	**23**	**24**	**25**	**26**	**27**	**47**	**46**	**45**	**44**	**43**	**42**	**41**	**31**	**32**	**33**	**34**	**35**	**36**	**37**
Sound	0	42	0	1	0	3	16	15	3	0	1	0	45	0	0	63	0	0	0	13	43	39	12	0	0	0	61	0
Dental caries	0	1	0	0	0	0	0	0	0	0	0	0	0	0	0	0	0	0	0	0	0	0	0	0	0	0	0	0
Not erupted	187	144	187	186	187	184	171	172	184	187	186	187	142	187	187	124	187	187	187	174	144	148	175	187	187	187	126	187

Bold numbers indicate the tooth kind by the WHO standard code. Almost no treated teeth were observed. Most of the permanent teeth were not erupted.

**Table 3:** Sensitivity of dental caries of deciduous teeth by item response theory (IRT) analysis.

		Item discrimination (*a*_*j*_)		Item difficulty (*b*_*j*_)
		Estimate	SE	
Maxillary	E	0.848	0.145	0.021
	D	−0.225	0.129	0.017
	C	1.632	0.195	0.038
	B	0.806	0.145	0.021
	A	0.441	0.139	0.019

Mandibular	E	−0.098	0.128	0.016
	D	−0.061	0.128	0.016
	C	2.291	0.265	0.070
	B	2.846	0.371	0.138
	A	2.690	0.370	0.137

Two parameter logistic models were applied for analysis. Item discrimination (*a*_*j*_) indicated the slope of the item response curve shown in Figure 2, and the item difficulty (*b*_*j*_) indicated the rising phase of the curve. Lower values indicate high sensitivity for dental varies (easy-to-be dental caries) in both item discrimination and item difficulty. Higher values indicate high tolerance for dental varies (hard-to-be dental caries).

**Table 4:** Results of multilevel modeling for the prevalence of dental caries at the tooth level.

		Coefficient	95% CI	*p* value
Intercept		1.125	0.168–2.082	0.024
Age		−0.127	−0.307–0.054	0.157
Male			Reference	
Female		0.077	−0.076–0.231	0.302
Maxillary	Molar		Reference	
	Canine	0.897	0.589–1.206	<0.001
	Anterior	0.063	−0.141–0.268	0.522
Mandibular	Molar	−0.277	−0.473–−0.082	0.008
	Canine	1.295	0.913–1.676	<0.001
	Anterior	1.607	1.224–1.991	<0.001

The fitness index of AIC is 145.718 and of BIC is 195.096. Each tooth was nested in each subjects. To analyze this hierarchal structure, multilevel analysis is indispensable. The coefficients showed the same tendency shown in IRT analysis in Table 2. Most eminent difference of these results from IRT was the adjustment of confoundings like age and gender.

## Data Availability

The data are available with the approval of the Ministry of Myanmar.
