# Corrigendum to “Prevalence of Dental Caries in 5- and 6-Year-Old Myanmar Children”

**DOI:** 10.1155/2019/6986412

**Published:** 2019-09-09

**Authors:** Yoshiaki Nomura, Khin Maung, Eint Min Kay Khine, Khin Myo Sint, May Phyo Lin, Min Khaing Win Myint, Thu Aung, Kaoru Sogabe, Ryoko Otsuka, Ayako Okada, Erika Kakuta, Wit Yee Wint, Masahide Uraguchi, Ryo Hasegawa, Nobuhiro Hanada

**Affiliations:** ^1^Department of Translational Research, Tsurumi University School of Dental Medicine, 2-1-3 Tsurumi, Tsurumi-ku, Yokohama 230-8501, Japan; ^2^Oral Health Division, Department of Medical Services, Ministry of Health and Sports, Naypyitaw, Myanmar; ^3^Department of Operative Dentistry, Tsurumi University School of Dental Medicine, 2-1-3 Tsurumi, Tsurumi-ku, Yokohama 230-8501, Japan; ^4^Department of Oral Bacteriology, Tsurumi University School of Dental Medicine, 2-1-3 Tsurumi, Tsurumi-ku, Yokohama 230-8501, Japan; ^5^Department of Pediatric Dentistry, Tokyo Medical and Dental University, 1-5-45 Yushima, Bunkyo-ku, Tokyo 113-8510, Japan; ^6^INGO Association of Dental Volunteers of Japan, 1-2693 Nishiminatomachi-dori, Cyuou-ku, Niigata 951-8026, Japan

In the article titled “Prevalence of Dental Caries in 5- and 6-Year-Old Myanmar Children” [1], it has been requested to remove two sentences from the Abstract and the Introduction, respectively, since the National Dental Survey has already started in Myanmar.

The updated Abstract and Introduction are as follows.

## 1. Abstract

In this study, we examined dental caries status of 187 school children located in the suburban area of Naypyidaw, capital of Myanmar, at the age of five and six and analyzed by the individual level and tooth level. Maxillary D and B were sensitive for dental caries almost at the same level. They were less sensitive than maxillary A. Mandibular A and B were tolerant for dental caries. Prevalence of dental caries in Myanmar children was still high. By applying item response theory and multilevel modeling, tooth level analysis can be implemented to confirm the tendency for sensitivity or tolerance for dental caries by the tooth level.

## 2. Introduction

Decline of the prevalence of dental caries is a global trend [1]. However, in developing countries, prevalence of dental caries is still at a high level [2–5]. In Myanmar, more than 7% of economic growth was achieved in 2018 due to ease of economic constraints. The IMF predicted that this trend will continue in the future. However, there are large disparities in living standards. Even in the suburbs of the capital city of Myanmar, the living infrastructure centering on agriculture, water supply, and sewerage is still not promoted. Some people do not have tooth-brushing habit, and fluoride contained tooth paste is not widely used. Even though evidence-based dental caries programs are available in developed countries [6–10], in most of the areas in Myanmar, these programs are not yet applicable. However, under these conditions, accumulating data on oral hygiene is important to establish national policies for improving oral hygiene. However, under these conditions, accumulation of the data of oral health is important to establish national policy for improvement of oral health.

In this study, we examined dental caries status of school children located in the suburban area of Naypyidaw, capital of Myanmar, at the age of five and six and analyzed by the individual level and tooth level. In addition, which kind of teeth is tended to be affected by dental caries was also examined by applying multilevel analysis.
